# Impact of Vitamin D Levels on Clinical Outcomes in SARS-CoV-2 Infections

**DOI:** 10.7759/cureus.78291

**Published:** 2025-01-31

**Authors:** Mouad Najih, Rihab Boussettine, Mohamed S El Kehel, Kawtar Nabil, Hasna Azmi, Hind Berradi, Moulay Mustapha Ennaji

**Affiliations:** 1 Laboratory of Virology, Oncology, Biosciences, and New Energies, Faculty of Sciences and Techniques Mohammedia, Mohammedia, MAR; 2 Laboratory of Healthcare Sustainable Development, Higher Institute of Nursing and Health Techniques (ISPITS), Casabanca, MAR

**Keywords:** covid-19, rt-pcr, sars-cov-2, symptoms, vitamin d

## Abstract

Background and aim: Since its emergence in 2019, SARS-CoV-2 has caused the global COVID-19 pandemic, presenting significant challenges for healthcare systems worldwide. Extensive research has focused on diagnostics, treatments, and vaccine development to combat the virus. Given the need for effective strategies to reduce infection rates, disease progression, and severity, this study aimed to investigate the potential role of vitamin D (25OHD) in mitigating the severity of COVID-19.

Methods: A cross-sectional study was conducted to evaluate the relationship between serum vitamin D levels and the severity of COVID-19 symptoms. Vitamin D levels were measured using the Roche Diagnostics Vitamin D assay in 100 SARS-CoV-2-infected patients confirmed by real-time polymerase chain reaction (PCR). Patients were categorized as symptomatic or asymptomatic, and the correlation between vitamin D levels and symptom severity was analyzed.

Results: The findings demonstrated that a significant proportion of symptomatic patients had vitamin D levels below 20 ng/mL. In contrast, approximately 25% of asymptomatic patients had vitamin D levels exceeding 30 ng/mL. Statistical analysis confirmed a significant association between low vitamin D levels and increased symptom severity (p=0.007).

Conclusion: This study suggests that vitamin D deficiency may contribute to the severity of COVID-19 symptoms. Vitamin D supplementation could potentially reduce the risk of severe disease. However, to confirm these findings and support these recommendations, further research, including randomized controlled trials and large-scale population studies, is necessary.

## Introduction

The well-being of individuals, particularly the immune system, relies significantly on various micronutrients, including vitamins C and D, as well as essential trace elements like zinc and selenium [1]. Amid the COVID-19 pandemic, there has been a surge in the use of vitamin D supplements, whether independently or under healthcare professional guidance. Ongoing research is exploring their potential efficacy through clinical trials [2]. Initiatives are being implemented to target interventions for individuals at high risk of vitamin D deficiency [3]. The primary motivation behind this effort to enhance vitamin levels lies in their ability to modulate the immune system, offering preventive or therapeutic effects against SARS-CoV-2 [4]. Worldwide studies have consistently highlighted vitamin D's role in the human body's natural defense system, achieved by stimulating the production of substances combating microorganisms, such as cathelicidins, defensins, and Interleukin-37 [5]. Additionally, 25-hydroxyvitamin D (25OHD) influences our response by managing crucial proinflammatory molecules like interleukin-6 (IL6), tumor necrosis factor-alpha (TNF-alpha), and interferon-gamma, along with regulating the activity of T helper 1 lymphocytes. Importantly, this regulation may be compromised in cases of vitamin D deficiency, but supplementation could potentially restore it [6,7].

Numerous studies have investigated the potential role of vitamin D in SARS-CoV infection. These inquiries have unveiled intricate interactions that contribute to protection against SARS-CoV-2 infection through a complex interplay between innate and adaptive immune responses [8,9]. The observed protective effects may be attributed, at least in part, to the ability of 25OHD to mitigate the cytokine storm associated with severe COVID-19 infection [10]. Notably, the active form of 25OHD not only inhibits inflammatory cytokines but also stimulates the expression of high levels of cytotoxic T-lymphocyte antigen 4 (CTLA-4) and Forkhead Box P3 (FoxP3), with the latter requiring the presence of interleukin-2 [11].

Health awareness approaches play a key role in improving the uptake of vitamin D supplements by targeting at-risk populations and promoting preventive behaviors in the face of deficiency. In addition, the integration of quality by design (QbD) enables the development of optimized and stable vitamin D formulations, ensuring increased efficacy and better bioavailability for improved clinical outcomes [12,13].

Recent studies on marketed vitamin D supplements show progress in innovative delivery systems, such as liposomes and emulgels, which are increasingly being studied to improve the absorption and bioavailability of vitamin D, particularly to overcome its low water solubility. For example, nanotechnological and polymeric formulations enable controlled and targeted release, optimizing the therapeutic effects of vitamin D while minimizing fluctuations in blood concentration. These advances offer promising solutions for ensuring more effective supplementation with vitamin D, which is essential for boosting immunity and preventing deficiency, particularly in diseases such as COVID-19 [14,15].

The objective of this study is to investigate the potential role of vitamin D in reducing the severity of COVID-19 symptoms, focusing on the relationship between serum 25OHD levels and the immune response triggered by COVID-19 infection, as well as examining the impact of clinical factors such as gender, age, and diabetes on symptom expression.

## Materials and methods

Patients 

This research is a cross-sectional study that included 100 individuals who tested positive for SARS-CoV-2 at the Anoual Laboratory Analysis in Casablanca, conducted between January 2021 and January 2022. The study was approved by the Ethics Committee of Biomedical Research in Casablanca, Morocco, and all participants signed informed consent for data use. The inclusion criteria consisted of individuals aged 18 to 80 years who tested positive for SARS-CoV-2 through real-time reverse transcription polymerase chain reaction (RT-PCR), with samples obtained from nasopharyngeal and pharyngeal swabs, and who had blood samples collected for the assessment of vitamin D levels. The enrollment technique involved the consecutive recruitment of eligible individuals who met the inclusion criteria during the study period. Demographic characteristics of the participants, such as age, sex, and health status, were recorded. The exclusion criteria included individuals with chronic diseases other than diabetes, pregnant women, patients undergoing chemotherapy or radiotherapy, those receiving immunosuppressive agents or systemic corticosteroids, and individuals with significant co-morbidities such as cancer, cardiovascular diseases, or autoimmune disorders. Additionally, participants currently involved in other clinical trials were excluded to ensure the reliability and accuracy of the findings.

The samples were accompanied by clinical and pathological parameters such as sex, age, fever, coughing, respiratory difficulties, headache, diarrhea, and diabetes (without taking into consideration the glycemic status). The study's specifics were clearly communicated to the participants, and informed consent was obtained from each individual enrolled.

Viral RNA extraction

Following the manufacturer's instructions, RNA extraction from nasopharyngeal and pharyngeal swab samples was performed using the automated Genrui 48 platform along with the Genrui Nucleic Acid Extraction assay. The resulting RNA extracts were then preserved at -80°C until their use.

Reverse transcriptase

Real-Time PCR 

Following RNA extraction, real-time PCR was conducted using 5 μL of RNA and the GeneFinderTM COVID-19 Plus kit. All procedures were carried out in accordance with relevant rules and regulations. Participants identified as positive in this study displayed cycle threshold (CT) values below 40 for all three genes (E, R, and N), confirming the presence of SARS-CoV-2 infection. The use of PCR to confirm SARS-CoV-2 infection is well-established and widely recognized for its accuracy. This study was conducted between January 2021 and January 2022, a period during which multiple variants of the virus circulated globally. The time frame is relevant as it may have coincided with particular COVID-19 waves or variant surges, potentially influencing the prevalence and characteristics of the infection in the study population.

Measurement of vitamin D levels 

The Roche Diagnostics Vitamin D assay (Cobas e411) was utilized to measure serum 25OHD levels. This method, designed for human serum and plasma, involves a three-step electrochemiluminescence immunoassay (ECLIA) process that takes about 27 minutes. Initially, a pretreatment reagent is used to release 25OHD from the vitamin D-binding protein (VDBP). Then, a complex forms between the sample and ruthenium-labeled VDBP, allowing for the incorporation of 25OHD. Finally, microparticles coated with biotin-labeled 25OHD are introduced, creating a complex that binds to a solid phase through biotin-streptavidin interaction. This method was chosen for its high reliability, precision, and reproducibility, as well as its widespread use in clinical laboratories. Quality control material from Roche Diagnostics ensured the accuracy of measurements, with coefficients of variation (CV) of 4.9% and 1.9% for concentrations of 43.3 nmol/L and 105 nmol/L, respectively. The assay has a broad linear measurement range and a low limit of detection, making it suitable for detecting both deficient and optimal levels of 25OHD. Serum 25OHD levels were categorized according to established clinical guidelines: vitamin D deficiency for levels below 20 ng/mL, inadequate vitamin D for 20-30 ng/mL, and optimal vitamin D for levels at or above 30 ng/mL.

Statistical analysis

Statistical data were processed using International Business Machines Statistical Package for the Social Sciences Statistics 28.0 for Windows (IBM SPSS 28 Statistics Windows, Armonk, NY: IBM Corp). The chi-square test was considered statistically significant when p≤0.05.

## Results

A cohort of 100 patients participated in this study, comprising 69 women and 31 men, with ages ranging from 20 to 92 years. All individuals were confirmed to be infected with SARS-CoV-2 through positive RT-PCR tests and exhibited the typical CT features of COVID-19 pneumonia. Among the participants, 84 (84%) had no chronic diseases, while 16 individuals (16%) had diabetes. Regarding symptom severity, 13% were asymptomatic (13 individuals), 46% were mildly symptomatic (46 individuals), and 41% were symptomatic (41 individuals). The vitamin D serum levels and clinical pathological data for all cases are detailed in Table 1.

**Table 1:** Clinical characteristics of patients included in the study (n=100)

Parameters		n (%)
Age	<40	27 (27%)
	40-60	40 (40%)
	>60	33 (33%)
Gender	Woman	69 (69%)
	Man	31 (31%)
Diabetic	No	84 (84%)
	Yes	16 (16%)
Vitamin D level	<20 ng/mL	33 (33%)
	20-30 ng/mL	43 (43%)
	>30 ng/mL	24 (24%)
Symptoms	Asymptomatic	13 (13%)
	Mild symptomatic	46 (46%)
	Symptomatic	41 (41%)

An important relationship between the symptomatic state and vitamin D levels was observed (p=0.007) with a significant association between these two factors. The majority of patients with symptomatic disease (64%) had vitamin D levels below 20 ng/mL, suggesting a potential link between lower vitamin D levels and the presence of symptoms. Interestingly, even among asymptomatic patients, 25% exhibited higher levels of vitamin D above 30 ng/mL, indicating a possible protective effect of adequate vitamin D levels. The statistical analysis confirmed the significance of this association between symptomatology and vitamin D levels (p=0.007) (Table 2). Additionally, adjustments for potential confounding factors such as age, gender, and underlying conditions (e.g., diabetes) were made in the analysis, ensuring a more accurate interpretation of the results.

**Table 2:** Clinical characteristics of the COVID-19 symptom severity Statistical analysis was performed using the Chi-square test. *Statistical significance with a p-value <0.05.

Parameters		Asymptomatic, n (%)	Mildly symptomatic, n (%)	Symptomatic, n (%)	P-value
Age	<40	3 (12%)	12 (44%)	12 (44%)	0.37
	40-60	4 (10%)	16 (40%)	20 (50%)	
	>60	6 (18 %)	18 (55%)	9 (27 %)	
Gender	Woman	9 (13%)	33 (48 %)	27 (39 %)	0.83
	Man	4 (13%)	13 (42 %)	14 (45 %)	
Diabetic	No	12 (15%)	41 (49%)	31 (36%)	0.15
	Yes	1 (6%)	5 (31%)	10 (63%)	
Vitamin D level	<20 ng/mL	2 (6%)	10 (30%)	21 (64%)	0.007*
	20-30 ng/mL	5 (11,6%)	26 (60,4%)	12 (28%)	
	>30 ng/mL	6 (25%)	10 (42%)	8 (33%)	

Furthermore, the investigation revealed no statistically significant correlations (P-value: 0.37, 0.83, 0.15) between age, gender, and diabetic status in relation to the presence of symptoms. Nevertheless, it is noteworthy that a higher percentage of diabetic participants (63%) manifested symptomatic presentations, as indicated in Table 2.

Moreover, we investigated to explore the relationship between Vitamin D levels, symptomatology, and diabetic status. The results revealed that non-diabetic patients tended to exhibit a lower incidence of symptoms compared to diabetic individuals, even when their vitamin D levels were similar, as indicated in Table 3. For instance, among diabetic patients experiencing symptoms, 80% had vitamin D levels below 20 ng/mL, whereas among non-diabetic patients with equivalent vitamin D levels, only 60% developed symptoms. The p-value (0.031) indicates a potential relationship between vitamin D levels and symptom development, particularly in diabetic patients (Table 3).

**Table 3:** Association of vitamin D levels with symptom severity and diabetic status Statistical analysis was performed using the chi-square test. *Statistical significance with a p-value <0.05.

Vitamin D		Asymptomatic	Mildly symptomatic	Symptomatic	P-value
Diabetic	<20 ng/mL	0 (%)	1 (20%)	4 (80%)	0.031*
	20-30 ng/mL	1 (11%)	4 (44,5%)	4 (44,5%)	
	>30 ng/mL	0 (%)	0 (%)	2 (100%)	
Non-diabetic	<20 ng/mL	3 (8%)	8 (32%)	17 (60%)	
	20-30 ng/mL	4 (14%)	23 (65%)	7 (21%)	
	>30 ng/mL	5 (20%)	10 (46,66%)	7(33,33%)	

## Discussion

Vitamin D is a crucial fat-soluble nutrient synthesized by the human body through exposure to ultraviolet B radiation from sunlight. Alternatively, it can be obtained through diet or supplements [16,17]. This essential vitamin plays a vital role in supporting immune function and regulating cell growth [18]. It activates immune cells, particularly in innate immunity, and enhances the production of antimicrobial peptides like cathelicidin, contributing to the defense against viral infections, such as human immunodeficiency virus HIV [19]. In adaptive immunity, vitamin D influences the differentiation of T helper cells (Th) and regulatory T cells (Treg), often promoting a T helper 2-type cytokine response that may be significant in autoimmune disorders [20-22]. Vitamin D deficiency is associated with reduced T cell proliferation, and its impact on early T cell signaling suggests a role in cytokine responses [23]. Regulatory T cells, crucial for priming cluster of differentiation 8 positive (CD8+) T cells and fostering effective immunological memory against infections, are also influenced by vitamin D [24]. It plays also a crucial role in respiratory health by regulating immune responses, with deficiency linked to an increased risk of respiratory infections such as tuberculosis and upper respiratory tract infections. Vitamin D enhances immune function through the activation of antimicrobial peptides, such as cathelicidin, and can improve lung function in individuals with asthma [25].

The COVID-19 pandemic has prompted exploration of vitamin D's potential role in the immune response against viral infections. Studies linking vitamin D levels to COVID-19 infection risk have found an association with vitamin D deficiency [26,27]. Although the exact protective mechanisms remain unclear, properties such as peptide production and regulation of pro-inflammatory cytokines, along with potential modulation of T helper 1 lymphocyte response, have been proposed [28,29]. Research suggests that vitamin D might mitigate the exaggerated immune response seen in severe COVID-19 cases and play a role in modulating ACE2 receptors, critical targets for SARS-CoV-2 entry into host cells [30,31]. The relationship between transmembrane protease, serine 2 (TMPRSS2), and angiotensin-converting enzyme 2 (ACE2) is critical to the progression of SARS-CoV-2 infection. TMPRSS2 plays a role in breaking down the spike protein, which aids in membrane fusion and helps the virus enter host cells, especially in the respiratory system. Conversely, ACE2 acts as the receptor for SARS-CoV-2, allowing the virus to enter cells. The interaction between TMPRSS2 and ACE2 is pivotal in determining the virus's infectivity and pathogenicity. Higher levels of TMPRSS2 expression may enhance viral entry and increase the severity of infection [32,33]. COVID-19 is more severe when hypertension is present due to the role of ACE2 in mediating inflammation [34]. This reaction is amplified by the virus when it binds to ACE2 receptors, increasing the probability of outcomes. Furthermore, treating hypertension with ACE inhibitors or angiotensin receptor blockers (ARBs) can increase ACE2 levels, making COVID-19 patients more vulnerable to SARS-CoV-2 infection and decreasing their prognosis. Various papers have noted that the prognosis of COVID-19 patients was not significantly affected by the use of hypertensive drugs [34,35]. Similarly, diabetes can impact the outcomes of COVID-19 by increasing the replication of the virus and impairing immune responses. It can also elevate blood sugar levels and lead to insulin resistance [36]. Furthermore, SARS-CoV-2 can infect endothelial cells through ACE2 receptors, resulting in endothelial dysfunction and hypercoagulation, which are characterized by decreased vasodilation, increased vascular permeability, and inflammation. The virus also causes an inflammatory response by releasing cytokines that harm the endothelium and generate a procoagulant environment. In severe situations, a cytokine storm worsens endothelial damage and disturbs the balance of procoagulant and anticoagulant substances, resulting in hypercoagulation [37]. Some papers have highlighted the link between thrombosis and COVID-19, such as a study comparing coronary thrombus features in STEMI patients with and without asymptomatic SARS-CoV-2 infection (ASAP). The ASAP group had a larger thrombus, a greater thrombus viral load, and a lower myocardial blush grade (MBG) than non-infected patients. They also demonstrated reduced left ventricular systolic function, greater troponin levels, and increased inflammatory markers [38]. Various studies like the one referenced consistently demonstrate a connection, between blood sugar levels and a negative outcome for COVID-19. Patients with high plasma glucose levels upon admission face a risk of developing illness or succumbing to the disease. However, managing blood sugar within the 24 hours of symptoms onset is associated with a reduced risk of severe illness or mortality indicating that COVID-19 patients with high blood sugar levels may benefit from early glycemic control regardless of whether they have been diagnosed with diabetes previously [39]. Hyperglycemia can also reduce vaccine efficacy by impairing immune function, including lymphocyte proliferation and cytokine production. It can also decrease antibody production and alter immune cell function, such as T cells. Additionally, hyperglycemia is associated with endothelial dysfunction, which can further weaken the immune response [40]. Indeed, the glycemic control status of individuals with type 2 diabetes (T2D) may have a major impact on the efficacy of COVID-19 immunization. According to new research, individuals with well-managed glycemia (HbA1c <7%) have been shown to respond more strongly to vaccinations than those with poorly controlled glycemia (HbA1c >7%). One of the main indicators of poorly managed T2D is hyperglycemia, which might compromise immune system performance and reduce the efficacy of the vaccine. For individuals with T2D, maintaining ideal glycemic control is crucial to boosting their immunological response to COVID-19 immunization. Optimizing vaccination efficacy and lowering the risk of breakthrough infections in this susceptible population requires the use of methods to enhance glycemic control, such as medication adherence and lifestyle adjustments [41].

Our research revealed a significant connection between vitamin D levels and COVID-19 symptoms (p=0.007). Most symptomatic patients had vitamin D levels below 20 ng/mL, suggesting a correlation between vitamin D levels and symptom manifestation. Interestingly, 25% of asymptomatic patients had vitamin D levels above 30 ng/mL, indicating a potential protective effect. Statistical analysis confirmed the significance of this relationship (p=0.007). These findings align with previous studies demonstrating a correlation between vitamin D levels and COVID-19 symptoms, such as the study by G. A. Sánchez Zuno, where patients with symptoms predominantly had vitamin D levels below 20 ng/mL [42].

Moreover, we explored the correlation between diabetes and the severity of COVID-19 symptoms. Our findings indicate that non-diabetic patients exhibit a lower symptom incidence compared to their diabetic counterparts. This observation aligns with a prior study conducted in Kuwait, which similarly reported that individuals with diabetes faced more severe COVID-19 outcomes than those without diabetes [43]. Diabetes, especially in the presence of hyperglycemia, significantly influences the severity of COVID-19 symptoms, possibly due to the association between hyperglycemia and elevated levels of IL-6 and IL-6 receptors, indicative of severe lung disease in COVID-19 patients. The connection between hyperglycemia and heightened IL-6 levels may amplify the inflammatory response, contributing to more severe symptoms [44,45].

The findings by Sait Gönen et al. in 2020 support the idea that administering vitamin D3 as a bolus supplement during or just before a COVID-19 infection is linked to less severe cases and increased survival rates, especially among vulnerable elderly individuals [46]. These results indicate that vitamin D supplements may help protect people at high risk of severe COVID-19, such as older adults and those with health conditions like diabetes and high blood pressure. Future studies should aim to determine the best dose and timing of vitamin D supplements to maximize their benefits, as well as assess their effectiveness against different COVID-19 variants. Additionally, researchers should explore how vitamin D might work together with other treatments to improve patient outcomes. 

This study suggests a potential link between vitamin D deficiency and more severe COVID-19 symptoms. While the results are promising and provide valuable insights into the potential role of vitamin D in modulating the immune response and the severity of COVID-19 symptoms, they show a significant association between low vitamin D levels and increased symptom severity, consistent with previous research. The study's limitations, such as a small sample size and lack of control for certain lifestyle factors, require further investigation. Larger, more comprehensive studies are needed to confirm these findings and explore the underlying mechanisms. Additionally, considering the impact of factors such as diet, sun exposure, and underlying health conditions on vitamin D levels and COVID-19 outcomes is crucial for a more comprehensive understanding. Therefore, the suggestion that vitamin D supplementation could be beneficial is based on correlation, and it should be clearly stated that further causal studies are needed to validate this hypothesis.

## Conclusions

We observed notable correlations between vitamin D levels and the onset of symptoms in patients with COVID-19. The recognized protective effects of Vitamin D against acute respiratory infections and its acknowledged safety support the idea of conducting targeted investigations into vitamin D levels among individuals with COVID-19 at various stages of disease severity. Vitamin D supplementation shows promise as a potentially effective, readily available, and well-tolerated treatment for COVID-19, a rapidly spreading condition currently lacking specific treatments. To establish the potential benefits of administering a vitamin D bolus to older adults during or just before infection to enhance or prevent COVID-19, further prospective studies, preferably employing an interventional approach, are essential.
